# Intelligence

**Published:** 1827-05

**Authors:** 


					INTELLIGENCE.
MONTHLY REPORT OF PREVALENT DISEASES.
Since our last Report we have met with several cases of Ague, and heard of
others, as well in London as in the vicinity. VVe suspect that Intermittent
Fever is becoming more frequent in the metropolis than it used to be formerly :
if so, on what may this depend? Affections of the Head have likewise been
rather more prevalent than usual: these have consisted chiefly of a febrile
paroxysm with very severe headache, but not running 011 to regular fever, if
met early by free purging atul local bleeding. Several cases of Apoplexy, fol-
lowed by hemiplegia, have presented themselves to our notice.
476 INTELLIGENCE.
Use of Bark in Iritis.
Sir,?-From a conviction that the following notice may be the means of
affording relief to many sufferers, I am desirous that it should be made known
as quickly and as extensively as possible: I therefore solicit you to give it a
placc in the first Number of your Journal.
I have the honour to be, Sir, your very obedient servant,
WM. WALLACE.
Gardner's Place, Dublin ; March 1827.
Of the numerous sequels which the prevailing fever of this country exhibits
during the state of convalescence, there are none more remarkable, nor more
frequent, than a peculiar inflammation of both the deep-seated and superficial
textures of the eye. This inflammation, from the symptoms which it pre-
sents, is entitled equally to the denomination of Iritis and Choroiditis. It
being my intention to publish a full account of this ophthalmic disease, so
soon as I shall have leisure to arrange my notes on the subject, I do not deem
it necessary to enlarge now upon this point. Those to whom this affection
may present itself will have no difficulty in distinguishing it by the history of
the case, conjoined with the existence of those symptoms which palpably de-
note an inflammation of the iris and choroid membrane.
As iritis, whether symptomatic or idiopathic, is generally treated by mer-
curials, with or without antiphlogistic remedies and counter-irritants, a similar
process of cure has been adopted in the iritis which succeeds fever. Such
were also the remediesl employed inthisdisease, until I found their inefficiency
on very many occasions; and, having been thus led to treat the affection upon
other principles, I acquired the knowledge of the singular property of Peru-
vian Bark to arrest this inflammatory action. The discovery has been made
known by me extensively in this city, partly among pupils during the course
of clinical instruction, and partly in conversation to professional friends con-
nected with hospitals; and it gives me great satisfaction to be able to state
that it has been fully confirmed by others. From Dr. Lendrick, physician
to Mercer's Hospital, and from Mr. Colles, surgeon to the Meath Hospital
and County of Dublin Infirmary, I have received communications, which will
be made known when I shall lay before the profession a full account of the
disease. At present it is sufficient to allude to these communications, as fully
confirming the efficacy of the remedy, even in those cases in which the mer-
curial treatment had failed.
For the same reasons that I refrain at present from any extended account
of Che symptoms of this disease, I shall not here enlarge either upon the causes
which led me to employ bark for its cure, or upon the views which I entertain
respecting the mode of action of this remedy. Discussions of this kind are
not necessary for the immediate object of this communication. That bark
should have the power of subduing a violent and disorganising inflammatory
action, will appear incompatible with the existing views of the action of this
remedy, and with the prevailing opinions of the nature of inflammation ; and
I know, from conversations with my professional brethren here, how difficultly
this treatment will be reconciled to the prevailing routine practice. Here it
may, however, be remarked, that it is not very long since the valuable disco-
very of the power of mercury to correct certain inflammatory actions of the
eye lias been generally admitted; nor was this discovery less in opposition to
the then existing views of many practitioners, than that is which I now make
known. I shall not disguise my confident hopes that this discovery of the
power of bark will hold a very high rank in importance. Its value will not
be confined to the disease in question. It is calculated to extend our views
of the powers of this remedy, and to improve our treatment of many forms
of inflammation. But, upon all these points,. 1 shall enlarge on a future
occasion.
Respecting the mode of exhibiting the bark, there is little to be said. It
Bark in Iritis?Neuralgia. 477
the inflammatory action be very violent, and the pain very distressing, I do
not venture to employ the remedy without previous purging and bleeding,
either topical or general. I am, however, by no means certain that this^ pre-
caution is necessary ; and I do not spend more than a day in this preliminary
treatment. While the patient is taking the bark, his bowels must be regu-
lated by any common gentle purgative. The bark in powder, and the sulphate
of quinine, I have found equally effectual. It is the pale bark which is vended
Jn general by druggists here. The dose of the powder which I employ is
about half a drachm three times a-day: this I direct to be taken in milk.
Whenever I use the Sulphate of Quinine, I prefer the form of solution, which
should be taken in such a quantity that the dose of the quinine shall be about
two grains three times a-day. Instances will occur in which larger doses must
be given than those which I have mentioned.
It may be proper to observe, that I do not assert that the disease in ques-
tion may not, on some occasions, be controlled by mercury, combined with
evacuants and counter-irritants; for I have known patients recover under
this treatment. But, in a very large proportion of these cases, such remedies
will not avail; and in many in which they may ultimately prove successful, a
prolonged treatment of some weeks may be required. Whereas, in a few
hours, I may say, the morbid action is suspended by the bark; and the organ,
which may have been for weeks in a state of disease, manifested by an almost
complete suspension of its functions and great alteration of structure, shall
he in a few days speedily and effectually restored to health. Whether this
peculiar form of ophthalmic disease, which is now, and has been for a long pe-
riod, so prevalent in Dublin, occurs in other countries; or whether it be even
a frequent occurrence in Ireland, except in the metropolis, I have not as yet
had any means of ascertaining. I therefore gladly take this opportunity of
soliciting, with a view to a farther publication, contributions from my profes-
sional brethren, not only in relation to the power of the remedy, but also
regarding the history and symptoms of the disease.
Neuralgia.?Dr. Darwall and Mr. Wickenden, highly respectable and
intelligent practitioners at Birmingham, have communicated to Mr. Hut-
chinson the following cases of Neuralgia:?
I. (By Mr. Wickenden.)?Mrs. Carless, zetatis sixty-four, Gough-street,
Birmingham, during two years has been gradually affected with severe pain
in her face. On the 16th of June, 18^3, its severity suddenly increased, and,
with a few minutes' intermission, continued four days. From her description
of the affected parts, the disease appeared to be confined to the ophthalmic
branch of the fifth pair of nerves. Calomel purges, with senna and salts, and
leeches to the face, were prescribed ; afterwards an emetic, succeeded by
large doses of opium, two grains of which were given every hour for six or
eight hours, without affording relief. She then took one drachm of the Sub-
carbonate of Iron every six hours, and felt perfectly relieved after the sixth
dose, and lias continued completely free from the disease up to this period,?
the 4th of November, 18'^S.
In further illustration of this very satisfactory case, it may be permitted to
remark that this lady suffered a pain of the most excruciating kind, concen-
trated in the snborbitar nerve of the right side of the face. This was aggra-
vated upon the slightest movement, and so fearful was she of touching it that
her face had remained unwashed for weeks together. Ot course, eating at all
times much increased it, and latterly she had eaten with a wooden spoon,?a
nietal one, if touching, causing such extreme aggravation of the pain."
Mr. Wickenden is a practitioner whose observation is too correct to render
a remark of his of no value. He observes, that formerly he used to give doses
of five grains of the iron, but that he never found any effect until he employed
the larger quantities now generally adopted.
II.?In the second case drawn up by Mr. Wickenden, the pain was seated
i" the upper lip, accompanied with frequent convulsive twitches, and was
478 INTELLIGENCE.
completely cured by drachm doses of the Carbonate of Iron. The pain in
this patient frequently returned, and was as frequently relieved by the Ferri
Subcarbon.
III.?The third case was marked by most severe pain in one side of the
head, particularly behind the ear, and recurring very frequently in the day.
After correcting the digestive organs without affording any relief, the Subcar-
bonate of Iron, in drachm doses, perfectly removed the disease.
I. (By Dr. Darwall.)?,e The first of these patients was Martha Foxley, a
young woman twenty years of age. The pains, which she described as ex-
ceedingly severe, came on regularly at five o'clock a.m. and three o'clock
p.m. They very much resembled those of neuralgia, having the same sudden
accessions and abatements, but were not particularly severe in the distribution
of the nerves, and were confined to the left half of the face and head, ceasing
exactly in the mesial line. Her health in other respects was tolerably good,
but the tongue was furred, and she occasionally had considerable indigestion.
After evacuating her bowels very freely of much offensive matter, she took
only one scruple of the Carbonate of Iron three times a-day for a week, when
she reported that she had no pain for three days. She now omitted the medi-
cine, but returned in a few days on account of a renewal of the attacks, when
half a drachm of the same medicine was ordered her three times a-day. In
another week the pain had again vanished; and, after continuing the medicine
for nine or ten days longer, she was a second time dismissed as cured. I have
not heard of her since, but I feel convinced that 1 should have seen her
again had she suffered any relapse."
II.?" June 9th, 1823. Mr. was , affected with syphilitic pains, com-
ing on with extreme severity in the night, and particularly affecting the right
knee, and accompanied with swelling both of the knees and ankles. On the
4tli of June, he was ordered half a drachm of the Ferri Subcarbon. three
times a-day. Since the 5th, he has had no pain of any consequence, and now
only complains of weakness. He slept last night six hours together, which he
had not done for several weeks before."
Southwell; April 4th.
Literary Notices.?Mr. Shaw has in the press an Essay an Aneurism of the
large "Vessels, with Remarks on the Proposal to revive the Operation of tying
the Artery beyond the Tumor, illustrated by plates and diagrams.
Dr. Hahrison has in the press, Pathological and Practical Observations
on Spinal Complaints, illustrated with Cases and engravings. Also an Inquiry
into the Origin and Cure of Distorted Limbs.
Mr. Curtis, the Surgeon to the Royal Dispensary for Diseases of the Ear,
has just published a Clinical Report of the Institution, from its commence-
ment to the present time ; with a Table of the number of Patients admitted,
cured, and relieved, showing the progressive increase and utility of the
Charity.
A vacancy has occurred in the office of Physician to the Middlesex Hospi-
tal, by the resignation of Dr. Southey. The election is to take place on the
24 th.
Sir E. Home has resigned the office of Surgeon to St. George's Hospital.
The election of his successor is fixed for the 11th of this month.
Royal College of Physicians.?The Lectures of the present year will he deli-
vered at the College, Pall Mall East, at four o'clock on each of the following
Wednesdays and Fridays.
Gulstonian Lectures?Dr. Watson. May 2, 4, 9.
Croonian Lectures?Dr. Yeats, on the Structure, Functions, and some
Diseases of the Colon. May 11, 16, IB.
Lumlcian Lectures?Dr. P. M. Latham. May 23, 25, 30.
Lecturcs on Materia Medica?Dr. Ager. June 1, 6, 8, 13, 15, 20.
480
METEOROLOGICAL JOURNAL,
From March 20th, to April '201 h, 1827.
By Messrs. Harris and Co. Mathematical Instrument Makers, 50, High Hoi bora.
20
21
22
23
24
25
26
27
28
29
30
SI
Mar.
1
2
3
4
5
6
7
8
9
10
i:
12
13
14
15
10
17
18
19
o
Barometer.
3
30.11
29.95
29.95
29.97
29.94
29.85
30.15
29.85
29.55
29.1 i
29.30
29.98
30.04
29.99
29 90
29.98
29.97
29.81
30.00
30.21
29.97
29.74
29.76
29.67
29.97
30.07
29.92
29.93
29.91
29.79
29.63
a
30.08
29.96
29.97
29.96
29.90
30.03
30.04
29.66
29.07
29.24
29.61
30.04
30.01
29.99
29.94
29.97
29.92
29.92
30.12
30.12
29.81
29.77
29.76
29.74
30.04
30.01
29.93
29.93
29.88
29.64
29.56
De Luc's
Hygroin.
Winds.
S\V
\V
\V
WNW
W
XV
N
ssw
\v
vvsw
WNW
N
wsw
wsvv
w
sw
s
sw
WNW
NE
SSE
WSW
ESE
SW
W
N
N
NNE
N
WNW
NE
Wvar,
W
W
WNW
W
WNW
!SW
WSW
sw
sw
NW
NW
SW
WSW
WSW
SSW
ESE
W
W
E
SW
ESE
SSW
SSW
NNW
NNW
NNW
N
SE
WNW
SE
Atmospheric Variations.
9 a.m. 2 p.m. 10 p.m
Cloudy
Fair
Cloudy
Fair
Cloudy
Cloudy
Fine
Fair
Cloudy
Hain
Cloudy
Fair
Fine
Cloudy
Fair
Fine
Cloudy
Fine
Hain
Fair

				

